# Supplementary material for: Transition to bio-based plastic packaging reveals complex climate–biodiversity trade-offs
Source: Nat Commun. 2026 Jan 31;17:3630. doi: 10.1038/s41467-026-69016-9 (PMC13096491; doi:10.1038/s41467-026-69016-9)
Supplement: Supplementary file 1 — Supplementary information [file 41467_2026_69016_MOESM1_ESM.docx]

***Supplementary information***

**Transition to bio-based plastic packaging reveals complex climate–biodiversity trade-offs**

Bilal Erradhouani^1,2^, Veronique Coma^2^, Guido Sonnemann^1^, Philippe Loubet^1^*

^1^Université de Bordeaux, CNRS, Bordeaux INP, ISM, UMR 5255, F-33400 Talence, France. ^2^Université de Bordeaux, CNRS, Bordeaux INP, LCPO, UMR 5629, F-33600 Pessac, France.

*e-mail: [philippe.loubet@u-bordeaux.fr](mailto:philippe.loubet@u-bordeaux.fr)

Table of contents

[Supplementary Fig. 1 | Life-cycle stages and system boundaries of fossil- and bio-based plastic packaging 1](#_Toc219470243)

[Supplementary Fig. 2 | Uncertainty analysis related to physical effects of MPs on marine biota associated with primary plastic packaging 2](#_Toc219470244)

[Supplementary Fig. 3 | Life-cycle material flows of fossil- and bio-based plastic packaging in Europe 3](#_Toc219470245)

[Supplementary Table 1 | Conversion and EOL parameters considered for packaging production in 2020 4](#_Toc219470246)

[Supplementary Table 2 | Conversion and EOL parameters considered for packaging production in 2050 4](#_Toc219470247)

[Supplementary Table 3 | Data source for the plastics packaging life cycles 5](#_Toc219470248)

[Supplementary Table 4 | Inventory data for the production of bio-PE from corn* 7](#_Toc219470249)

[Supplementary Table 5 | Inventory data for the production of bio-PET (30%) from corn* 8](#_Toc219470250)

[Supplementary Table 6 | Inventory data for the production of PLA from corn* 9](#_Toc219470251)

[Supplementary Table 7 | Inventory data for the production of PLA from corn stover* 10](#_Toc219470252)

[Supplementary Table 8 | Inventory data for the production of PLA from sugarcane* 11](#_Toc219470253)

[Supplementary Table 9 | Inventory data for the production of PHB from sugarcane* 12](#_Toc219470254)

[Supplementary Table 10 | Parameters and assumptions used to estimate greenhouse gas emissions from landfilling of bio-based plastic packaging (1 kg) 13](#_Toc219470255)

[Supplementary Table 11 | Parameters and assumptions used to estimate greenhouse gas emissions from composting of bio-based plastic packaging (1 kg) 14](#_Toc219470256)

[Supplementary Table 12 | Parameters and assumptions used to estimate greenhouse gas emissions from marine littering of plastic packaging (1 kg) 15](#_Toc219470257)

[Supplementary Table 13 | Parameters for the characterization of microplastics impacts in LCA 16](#_Toc219470258)

[Supplementary Table 14 | Assumptions used to assess geographical variability in feedstock and energy for polymer production 17](#_Toc219470259)

[Supplementary Table 15 | Plastics demand in the European Union* 18](#_Toc219470260)

[Supplementary Table 16 | Index 19](#_Toc219470261)

[Supplementary Methods 20](#_Toc219470262)

[Supplementary References 22](#_Toc219470263)

**
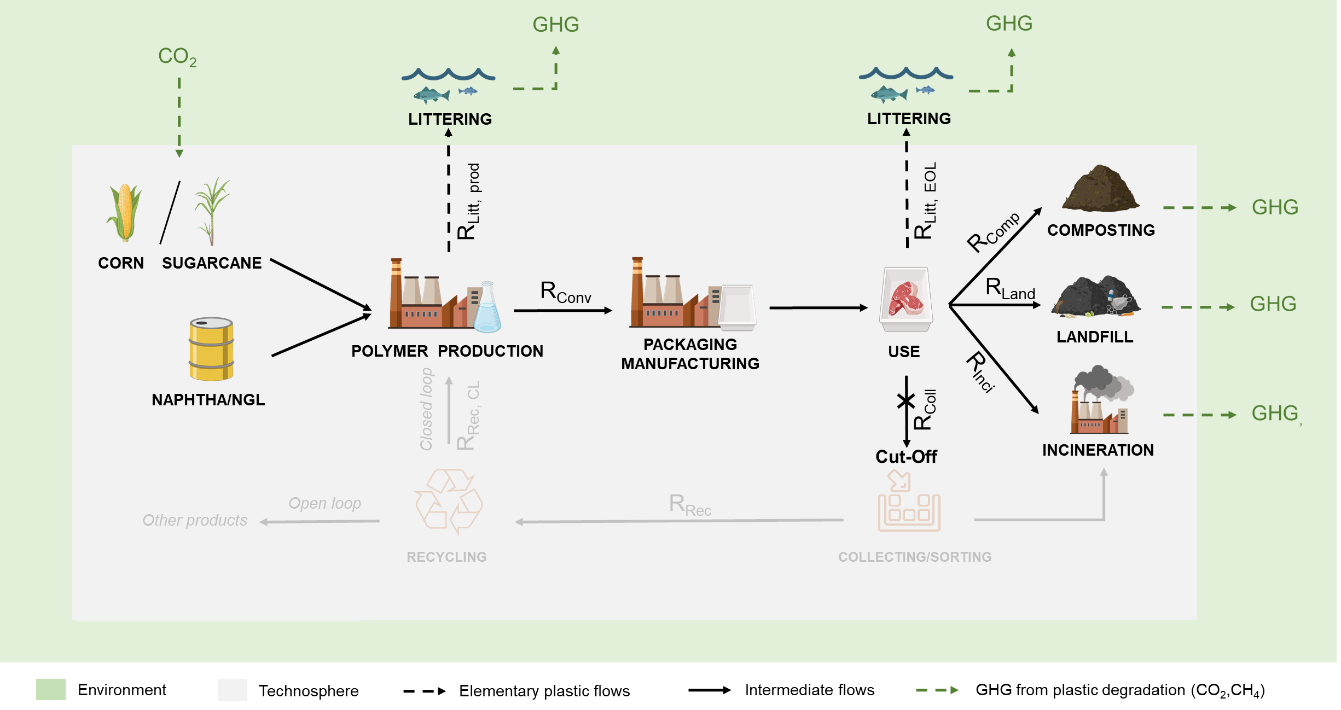
****Supplementary Fig. 1 | Life-cycle stages and system boundaries of fossil- and bio-based plastic packaging.** Life-cycle stages considered for the production of fossil- and bio-based plastic packaging, from cradle to grave, along with the associated conversion rates. Intermediate flows represent exchanges of materials and energy within the technosphère. Elementary flows correspond to exchanges between the technosphere and the environment. Only selected elementary flows are shown, focusing on potential sources of biogenic carbon sequestration or GHG emissions across the life cycle, as well as on the main pathways leading to plastic debris leakages. A cut-off approach was applied to the end-of-life recycling of packaging materials, meaning that the impacts associated with material collection and recycling processes were excluded from the system boundaries (burden-free). The conversion (R_Conv_), collection (R_Coll_), end-of-life (R_Comp_, R_Land_, R_Inci_), and plastic leakage (R_Litt_) rates are provided in Supplementary Table 1. Created in BioRender. Erradhouani, B. (2026) <https://BioRender.com/suv9dat>


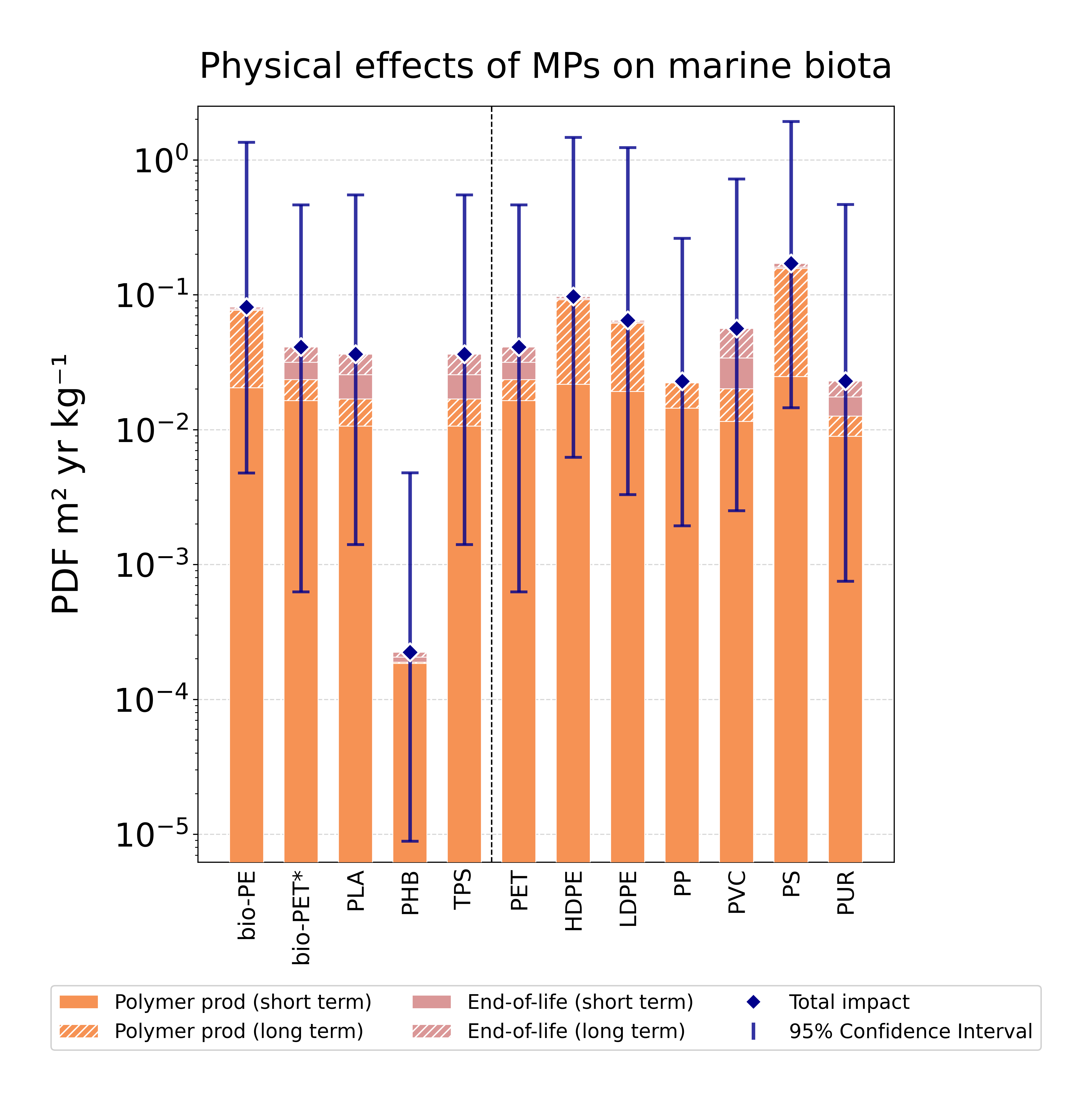


**Supplementary Fig. 2 | Uncertainty analysis related to physical effects of MPs on marine biota associated with primary plastic packaging**. Impacts are evaluated on a logarithmic scale. The uncertainty analysis, performed using Monte Carlo simulations, was adapted from data provided by Saadi et al. (2025). The resulting values, expressed in PDF m² yr (Potentially Disappeared Fraction of species over a given area and time), span approximately one order of magnitude above and below the central estimates for all polymers.


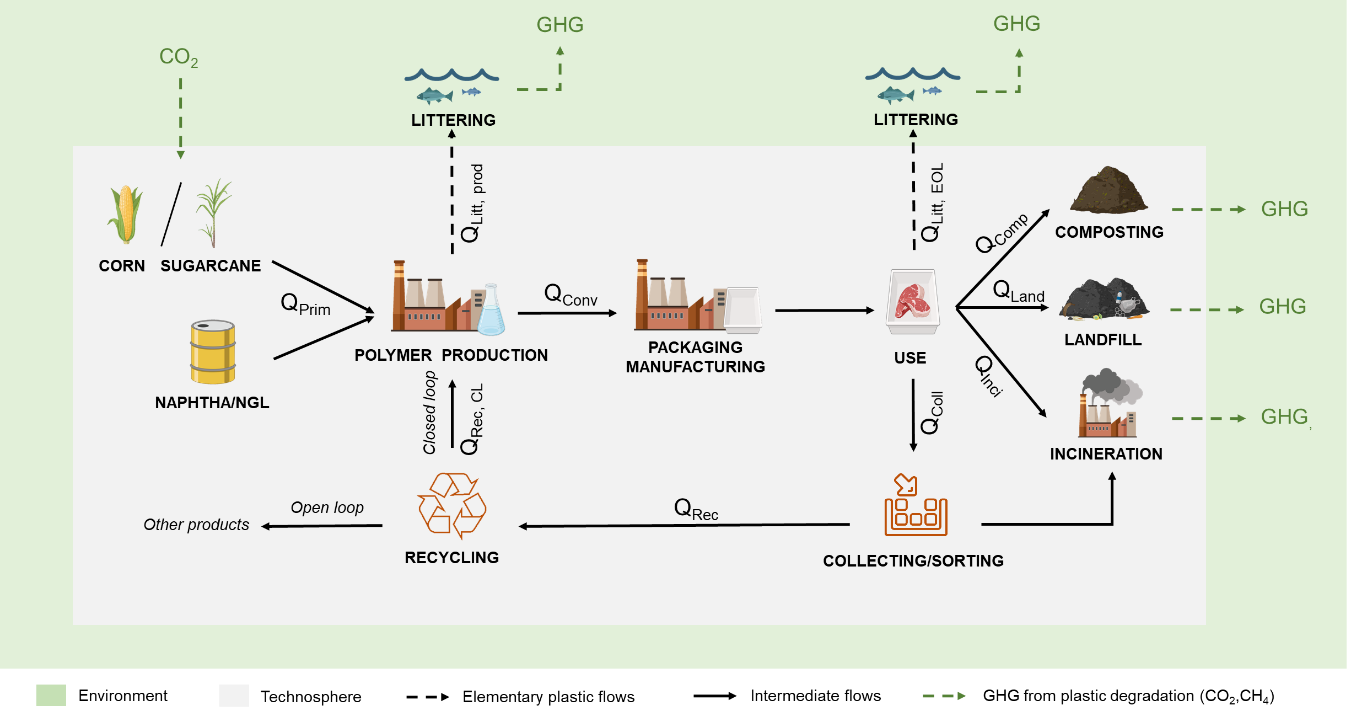


**Supplementary Fig. 3 | Life-cycle material flows of fossil- and bio-based plastic packaging in Europe**. Life-cycle stages considered for the production of fossil- and bio-based plastic packaging, from cradle to grave, together with the associated material quantities. Quantities (Q) were estimated based on available conversion rates and projected packaging demand in Europe (see supplementary tables 1, 2, 13 and 14 and supplementary equations (4)-(14)). Created in BioRender. Erradhouani, B. (2026) <https://BioRender.com/suv9dat>

# **Supplementary Table 1 | Conversion and EOL parameters considered for packaging production in 2020**

| **Polymer** | **R_Conv_** | **R_Litt, prod_** | **R_Inci_** | **R_Land_** | **R_Comp_** | **R_Litt, EOL_** | **R_Coll_*** | **R_Rec_*** | **R_Rec, CL_** |
| --- | --- | --- | --- | --- | --- | --- | --- | --- | --- |
| HDPE - LDPE | 1.06 | 0.0001 | 0.31 | 0.31 | 0 | 0.01 | 0.38 | 0.63 | 1 |
| PET | 1.06 | 0.0001 | 0.21 | 0.21 | 0 | 0.01 | 0.57 | 0.64 | 1 |
| PP | 1.06 | 0.0001 | 0.37 | 037 | 0 | 0.01 | 0.24 | 0.79 | 1 |
| PS | 1.06 | 0.0001 | 0.46 | 0.46 | 0 | 0.01 | 0.07 | 0.78 | 1 |
| PUR | 1.06 | 0.0001 | 0.47 | 0.47 | 0 | 0.01 | - | - | 1 |
| PVC | 1.06 | 0.0001 | 0.50 | 0.50 | 0 | 0.01 | - | - | 1 |
| Bio-PE | 1.06 | 0.0001 | 0.31 | 0.31 | 0 | 0.01 | 0.38 | 0.63 | 1 |
| Bio-PET | 1.06 | 0.0001 | 0.21 | 0.21 | 0 | 0.01 | 0.57 | 0.71 | 1 |
| PLA | 1.06 | 0.0001 | 0.42 | 0.42 | 0.16 | 0.01 | - | - | 1 |
| PHB | 1.06 | 0.0001 | 0.42 | 0.42 | 0.16 | 0.01 | - | - | 1 |
| TPS | 1.06 | 0.0001 | 0.42 | 0.42 | 0.16 | 0.01 | - | - | 1 |

*****Adapted from Zheng et al. (2019)^1^; Cimpan et al. (2021)^2^; eunomia (2020)^3^ and ICIS (2022)^4^.

# **Supplementary Table 2 | Conversion and EOL parameters considered for packaging production in 2050**

| **Polymer** | **R_Conv_** | **R_Litt, prod_** | **R_Inci_** | **R_Land_** | **R_Comp_** | **R_Litt, EOL_** | **R_Coll_*** | **R_Rec_*** | **R_Rec, CL_** |
| --- | --- | --- | --- | --- | --- | --- | --- | --- | --- |
| HDPE - LDPE | 1.06 | 0.0001 | 0.31 | 0.31 | 0 | 0.01 | 0.70 | 0.63 | 1 |
| PET | 1.06 | 0.0001 | 0.21 | 0.21 | 0 | 0.01 | 0.69 | 0.64 | 1 |
| PP | 1.06 | 0.0001 | 0.37 | 037 | 0 | 0.01 | 0.56 | 0.79 | 1 |
| PS | 1.06 | 0.0001 | 0.46 | 0.46 | 0 | 0.01 | 0.57 | 0.78 | 1 |
| PVC | 1.06 | 0.0001 | 0.50 | 0.50 | 0 | 0.01 | 0.59 | 0.63 | 1 |
| Bio-PE | 1.06 | 0.0001 | 0.23 | 0.06 | 0 | 0.01 | 0.70 | 0.63 | 1 |
| Bio-PET | 1.06 | 0.0001 | 0.31 | 0.06 | 0 | 0.01 | 0.62 | 0.71 | 1 |
| PLA | 1.06 | 0.0001 | 0.14 | 0.06 | 0.2 | 0.01 | 0.70 | 0.63 | 1 |
| PHB | 1.06 | 0.0001 | 0.14 | 0.06 | 0.2 | 0.01 | 0.70 | 0.63 | 1 |
| TPS | 1.06 | 0.0001 | 0.14 | 0.06 | 0.2 | 0.01 | 0.70 | 0.63 | 1 |

*****Adapted from Zheng et al. (2019)^1^.

# **Supplementary Table 3 | Data source for the plastics packaging life cycles**

| **Polymer type** | **Stage** | **Unit process** | **Source** |
| --- | --- | --- | --- |
| PET, HDPE, LDPE, PP, PS, PUR, PVC | Feedstock | market for naphtha \| RoW | ecoinvent 3.10 |
| PET, HDPE, LDPE, PP, PS, PUR, PVC | Feedstock | market for natural gas liquids \| GLO | ecoinvent 3.10 |
| bio-PE/bio-PET/TPS | Feedstock | market group for maize grain \| US | ecoinvent 3.10 |
| PHB | Feedstock | sugarcane production \| BR-MT | ecoinvent 3.10 |
| PLA | Feedstock | market group for maize grain \| US  market group for maize stover \| US | ecoinvent 3.10  Agribalyse 3.1 |
| PHB | Feedstock | sugarcane production \| BR-MT | ecoinvent 3.10 |
| PET | Polymer production | market for polyethylene terephthalate, granulate, amorphous \| GLO | ecoinvent 3.10 |
| HDPE | Polymer production | market for polyethylene, high density, granulate \| GLO | ecoinvent 3.10 |
| LDPE | Polymer production | market for polyethylene, low density, granulate \| GLO | ecoinvent 3.10 |
| PP | Polymer production | market for polypropylene, granulate \| GLO | ecoinvent 3.10 |
| PS | Polymer production | market for polystyrene, general purpose \| GLO | ecoinvent 3.10 |
| PUR | Polymer production | market for polyurethane, flexible foam \| RoW | ecoinvent 3.10 |
| PVC | Polymer production | market for polyvinylchloride, bulk polymerised \| GLO | ecoinvent 3.10 |
| Bio-PE | Polymer production | Harmonization procedure | ecoinvent 3.10; Vural et al. (2021)^5^; Benavides et al. (2020)^6^ |
| Bio-PET | Polymer production | Harmonization procedure | ecoinvent 3.10; Vural et al. (2021)^5^; Chen et al. (2016)^7^; García-Velásquez C. et al. (2022)^8^ |
| PLA | Polymer production | Harmonization procedure | ecoinvent 3.10; Benavides et al. (2020)^6^ |
| PHB | Polymer production | Harmonization procedure | ecoinvent 3.10; Harding et al. (2007)^9^ |
| TPS | Polymer production | polyester-complexed starch biopolymer production \| RoW | ecoinvent 3.10 |
| All polymers | Conversion | market for extrusion of plastic sheets and thermoforming, inline \| GLO | ecoinvent 3.10 |
| PET/bio-PET | Landfill | treatment of waste polyethylene terephthalate, sanitary landfill \| RoW | ecoinvent 3.10 |
| HDPE/LDPE/bio-PE | Landfill | treatment of waste polyethylene, sanitary landfill \| RoW \| waste polyethylene | ecoinvent 3.10 |
| PP | Landfill | treatment of waste polypropylene, sanitary landfill \| RoW | ecoinvent 3.10 |
| PS | Landfill | treatment of waste polystyrene, sanitary landfill \| RoW | ecoinvent 3.10 |
| PUR | Landfill | treatment of waste polyurethane, sanitary landfill \| RoW | ecoinvent 3.10 |
| PVC | Landfill | treatment of waste polyvinylchloride, sanitary landfill \| RoW | ecoinvent 3.10 |
| PLA/PHB/TPS | Landfill | Harmonization procedure | ecoinvent 3.10; Benavides et al. (2020)^6^ |
| PET/bio-PET | Incineration | treatment of waste polyethylene terephthalate, municipal incineration \| GLO | ecoinvent 3.10 |
| HDPE/LDPE/bio-PE | Incineration | treatment of waste polyethylene, municipal incineration \| GLO | ecoinvent 3.10 |
| PP | Incineration | treatment of waste polypropylene, municipal incineration \| GLO | ecoinvent 3.10 |
| PS | Incineration | treatment of waste polystyrene, municipal incineration \| GLO | ecoinvent 3.10 |
| PVC | Incineration | treatment of waste polyvinylchloride, municipal incineration \| GLO | ecoinvent 3.10 |
| PUR | Incineration | treatment of waste polyurethane, municipal incineration \| GLO | ecoinvent 3.10 |
| PLA/PHB/TPS | Incineration | (adapted from) treatment of waste plastic, mixture, municipal incineration \| waste plastic, mixture | ecoinvent 3.10 |
| PLA/PHB/TPS | Composting | Harmonization procedure | ecoinvent 3.10; Gastaldi et al. (2024)^10^; Benavides et al. (2020)^6^ |
| PE/PET/PP/PS/bio-PE/bio-PET | Collecting/sorting/Recycling | Adapted rates from literature | Cimpan et al. (2021)^2^; ICIS (2022)^4^; eunomia (2020)^3^ |
| PE/bio-PE | Recycling | pelletising of polyethylene \| RER | ecoinvent 3.11 |
| PET/bio-PET/PLA/PHB/TPS | Recycling | pelletising of polyethylene terephthalate \| RER | ecoinvent 3.11 |
| PP | Recycling | pelletising of polypropylene \| RER | ecoinvent 3.11 |
| PS | Recycling | pelletising of polystyrene \| RER | ecoinvent 3.11 |
| PVC | Recycling | pelletising of polyvinylchloride \| RER | ecoinvent 3.11 |
| PE/PET/PP/PS/bio-PE/bio-PET | Recycling, closed loop | Assumption |  |
| All polymers | Littering | Adapted rates from literature | PLP (2020)^11^; Loubet et al. (2022)^12^; Cimpan et al. (2021)^2^ |

# **Supplementary Table 4 | Inventory data for the production of bio-PE from corn***

| **Category of flow** | **Flow** | **Input/Output** | **Unit process (ecoinvent 3.10 process)** | **Quantity** | **Unit** |
| --- | --- | --- | --- | --- | --- |
| Feedstock | Corn | Input | market group for maize grain \| US | 6.2 | kg |
| Chemicals | Lime | Input | market for lime \| RER | 0.006 | kg |
|  | Sulfuric acid | Input | market for sulfuric acid \| RER | 0.029 | kg |
|  | Sodium hydroxide | Input | market for sodium hydroxide, without water, in 50% solution state \| RER | 0.021 | kg |
|  | Water | Input | market for tap water \| Europe without Switzerland | 9.41 | kg |
|  | Soda ash | Input | market for soda ash, light \| RER | 0.077 | kg |
|  | (NH4)2SO4 | Input | market for ammonium sulfate \| RER | 0.021 | kg |
|  | Nitrogen fertilizer | Input | market group for inorganic nitrogen fertiliser, as N | 0.021 | kg |
|  | Ammonia | Input | ammonia production, steam reforming, liquid \| RER w/o RU | 0.013 | kg |
|  | Urea | Input | market for urea \| RER | 0.0024 | kg |
|  | Water cooling | Input | Water, cooling, unspecified natural origin | 110 | kg |
| Energy | Heat | Input | market group for heat, district or industrial, natural gas \| GLO | 38.506 | MJ |
|  | Electricity | Input | market group for electricity, low voltage \| GLO | 1.62 | kWh |
|  | Heavy fuel | Input | market for heavy fuel oil \| Europe without Switzerland | 0.037 | kg |
| Infra- structure | Chemical factory | Input | market for chemical factory, organics \| GLO | 4E-10 | item |
|  | Ethanol fermentation plant | Input | ethanol fermentation plant construction \| RoW | 1.067E-09 | item |
| Waste | Wastewater | Output | market for wastewater, average \| Europe without Switzerland | 0.0180 | m3 |
| **Reference flow** | **Bio-PE resin** | **Output** |  | **1** | **kg** |

*Adapted from García-Velásquez C. et al. (2022)^8^, Chen et al. (2016)^7^, Vural et al. (2021)^5^, Benavides et al. (2020)^6^ and ecoinvent 3.10.

# **Supplementary Table 5 | Inventory data for the production of bio-PET (30%) from corn***

| **Category of flow** | **Flow** | **Input/ Output** | **Unit process (ecoinvent 3.10 process)** | **Quantity** | **Unit** |
| --- | --- | --- | --- | --- | --- |
| Feedstock | Corn | Input | market group for maize grain \| US | 1.76 | kg |
| Chemicals | Lime | Input | market for lime \| RER | 0.002 | kg |
|  | Cobalt | Input | market for cobalt \| GLO | 0.0002 | kg |
|  | Chemical, inorganic | Input | market for chemical, inorganic \| GLO | 0.0005 | kg |
|  | Chemical, organic | Input | market for chemical, organic \| GLO | 0.007 | kg |
|  | Oxygen liquid | Input | market for oxygen, liquid \| RER | 0.2161 | kg |
|  | Compressed air | Input | market for compressed air, 600 kPa gauge \| RER | 0.303 | kg |
|  | p-xylene | Input | market for p-xylene \| RER | 0.576 | kg |
|  | Sulfuric acid | Input | market for sulfuric acid \| RER | 0.0083 | kg |
|  | Sodium hydroxide | Input | market for sodium hydroxide, without water, in 50% solution state \| RER | 0.0184 | kg |
|  | Water | Input | market for tap water \| Europe without Switzerland | 4.82 | kg |
|  | Soda ash | Input | market for soda ash, light \| RER | 0.022 | kg |
|  | (NH_4_)_2_SO_4_ | Input | market for ammonium sulfate \| RER | 0.0059 | kg |
|  | Nitrogen fertilizer | Input | market group for inorganic nitrogen fertiliser, as N | 0.0059 | kg |
|  | Ammonia | Input | ammonia production, steam reforming, liquid \| RER w/o RU | 0.0037 | kg |
|  | Urea | Input | market for urea \| RER | 0.00067 | kg |
|  | Water cooling | Input | Water, cooling, unspecified natural origin | 39.33 | kg |
| Energy | Heat | Input | market group for heat, district or industrial, natural gas \| GLO | 17.83 | MJ |
|  | Electricity | Input | market group for electricity, low voltage \| GLO | 0.84 | kWh |
|  | Heavy fuel | Input | market for heavy fuel oil \| Europe without Switzerland | 0.0107 | kg |
|  | Steam | Input | market for steam, in chemical industry \| RER | 0.94 | kg |
| Infra- structure | Chemical factory | Input | market for chemical factory, organics \| GLO | 1.1E-09 | item |
|  | Ethanol fermentation plant | Input | ethanol fermentation plant construction \| RoW | 3.04E-10 | item |
| Waste | Wastewater | Output | market for wastewater, average \| Europe without Switzerland | 0.0180 | m3 |
|  | Waste plastic | Output | market group for waste plastic, mixture | 0.00231 | kg |
| **Reference flow** | **Bio-PET(30%) resin** | **Output** |  | **1** | **kg** |

*Adapted from García-Velásquez C. et al. (2022)^8^, Chen et al. (2016)^7^, Vural et al. (2021)^5^ and ecoinvent 3.10.

# **Supplementary Table 6 | Inventory data for the production of PLA from corn***

| **Category of flow** | **Flow** | **Input/Output** | **Unit process (ecoinvent 3.10 process)** | **Quantity** | **Unit** |
| --- | --- | --- | --- | --- | --- |
| Feedstock | Corn | Input | market group for maize grain \| US | 2.136 | kg |
| Chemicals | Lime | Input | market for lime \| RER | 0.79 | kg |
|  | Sulfuric acid | Input | market for sulfuric acid \| RER | 0.25 | kg |
|  | Sodium chloride, powder | Input | market for sodium chloride, powder \| GLO | 0.11 | kg |
|  | Water | Input | market for tap water \| Europe without Switzerland | 3.2 | kg |
| Energy | Heat | Input | market group for heat, district or industrial, natural gas \| GLO | 18.7 | MJ |
|  | Electricity | Input | market group for electricity, low voltage \| GLO | 1.85 | kWh |
| Infrastructure | Chemical factory | Input | market for chemical factory, organics \| GLO | 4E-10 | item |
| Waste | Hazardous waste | Output | market for hazardous waste, for incineration \| Europe without Switzerland | 0.0064 | kg |
|  | Wastewater | Output | market for wastewater, average \| Europe without Switzerland | 0.003 | m3 |
| **Reference flow** | **PLA resin** | **Output** |  | **1** | **kg** |

*Adapted from Vink et al. (2015)^13^ and ecoinvent 3.10.

# **Supplementary Table 7 | Inventory data for the production of PLA from corn stover***

| **Category of flow** | **Flow** | **Input/Output** | **Unit process (ecoinvent 3.10 process)** | **Quantity** | **Unit** |
| --- | --- | --- | --- | --- | --- |
| Feedstock | Corn stover | Input | maize stover, irrigated, at farm (adapted from WFLDB) \| US | 2.7 | kg |
| Chemicals | Lime | Input | market for lime \| RER | 0.85 | kg |
|  | Sulfuric acid | Input | market for sulfuric acid \| RER | 1.4 | kg |
|  | Enzyme | Input | enzyme production, bacterial alpha-amylase (adapted from WFLDB) \| GLO | 0.13 | kg |
|  | Whey | Input | market for whey \| GLO | 0.11 | kg |
|  | Ammonia | Input | ammonia production, steam reforming, liquid \| RER w/o R | 0.03 | kg |
|  | Water | Input | market for tap water \| Europe without Switzerland | 9.5 | kg |
| Energy | Heat | Input | market group for heat, district or industrial, natural gas \| GLO | 21.25 | MJ |
|  | Steam | Input | market for steam, in chemical industry \| RER | 3.8 | kg |
|  | Electricity | Input | market group for electricity, low voltage \| GLO | 0.26 | kWh |
| Infrastructure | Chemical factory | Input | market for chemical factory, organics \| GLO | 4E-10 | item |
| Waste | Inert waste | Output | market for inert waste \| RER | 0.94 | kg |
|  | Solid waste | Output | market group for municipal solid waste \| RER | 0.56 | kg |
|  | Biowaste | Output | market for biowaste \| RoW | 0.55 | kg |
|  | Wastewater | Output | market for wastewater, average \| Europe without Switzerland | 0.009 | m3 |
| **Reference flow** | **PLA resin** | **Output** |  | **1** | **kg** |

*Adapted from Ioannidou et al. (2022)^14^ and ecoinvent 3.10.

# **Supplementary Table 8 | Inventory data for the production of PLA from sugarcane***

| **Category of flow** | **Flow** | **Input/Output** | **Unit process (ecoinvent 3.10 process)** | **Quantity** | **Unit** |
| --- | --- | --- | --- | --- | --- |
| Feedstock | Sugarcane | Input | sugarcane production \| BR-MT | 11.5 | kg |
| Chemicals | Lime | Input | market for lime \| RER | 0.85 | kg |
|  | Sulfuric acid | Input | market for sulfuric acid \| RER | 1.35 | kg |
|  | Whey | Input | market for whey \| GLO | 0.11 | kg |
|  | Water | Input | market for tap water \| Europe without Switzerland | 3.2 | kg |
| Energy | Heat | Input | market group for heat, district or industrial, natural gas \| GLO | 22.33 | MJ |
|  | Electricity | Input | market group for electricity, low voltage \| GLO | 1.29 | kWh |
| Infrastructure | Chemical factory | Input | market for chemical factory, organics \| GLO | 4E-10 | item |
| Waste | Inert waste | Output | market for inert waste \| RER | 0.94 | kg |
|  | Biowaste | Output | market for biowaste \| RoW | 0.09 | kg |
|  | Wastewater | Output | market for wastewater, average \| Europe without Switzerland | 0.009 | m3 |
| **Reference flow** | **PLA resin** | **Output** |  | **1** | **kg** |

*Adapted from Ioannidou et al. (2022)^14^ and ecoinvent 3.10.

# **Supplementary Table 9 | Inventory data for the production of PHB from sugarcane***

| **Category of flow** | **Flow** | **Input/Output** | **Unit process (ecoinvent 3.10 process)** | **Quantity** | **Unit** |
| --- | --- | --- | --- | --- | --- |
| Feedstock | Sugarcane | Input | sugarcane production \| BR-MT | 16.02 | kg |
| Chemicals | Quicklime | Input | market for quicklime, milled, loose \| RoW | 0.013 | kg |
|  | Sulfuric acid | Input | market for sulfuric acid \| RER | 0.0056 | kg |
|  | Calcium chloride | Input | market for calcium chloride \| RER | 0.0023 | kg |
|  | Na_2_HPO_4_ | Input | sodium phosphate production \| RER | 0.003 | kg |
|  | Chemical, inorganic | Input | market for chemical, inorganic \| GLO | 0.01 | kg |
|  | (NH_4_)_2_SO_4_ | Input | market for ammonium sulfate \| RER | 0.0148 | kg |
|  | Inorganic potassium fertiliser | Input | inorganic potassium fertiliser, as K2O to generic market for organic potassium fertiliser, as K2O \| GLO | 0.0209 | kg |
|  | Hydrogen peroxide | Input | market for hydrogen peroxide, without water, in 50% solution state \| RER | 0.053 | kg |
| Energy | Heat | Input | market group for heat, district or industrial, natural gas \| GLO | 14.37 | MJ |
|  | Electricity | Input | market group for electricity, low voltage \| GLO | 2.23 | kWh |
| Infrastructure | Chemical factory | Input | market for chemical factory, organics \| GLO | 4E-10 | item |
| **Reference flow** | **PLA resin** | **Output** |  | **1** | **kg** |

*****Adapted from Harding et al. (2007)^9^ and ecoinvent 3.10.

# **Supplementary Table 10 | Parameters and assumptions used to estimate greenhouse gas emissions from landfilling of bio-based plastic packaging (1 kg)**

|  | **PLA** | **PHB** | **TPS** |
| --- | --- | --- | --- |
| Formula | C_3_H_4_O_2_ | C_9_H_14_O_4_ | C_6_H_12_O_5_ |
| MM_polymer_ | 72 | 194 | 164 |
| Carbon content | 0.5 | 0.56 | 0.44 |
| CO_2_ uptake | 1.83 | 2.04 | 1.61 |
| % biodeg (100yrs)** | 40 | 42.5 | 40 |
| [CH_4_] (%) | 50 | 50 | 50 |
| CH_4_ collection efficiency (%) | 75 | 75 | 75 |
| CH_4_ oxidation (%) | 36 | 36 | 36 |
| CO_2_^s^ (kgCO_2_) | 1.10 | 1.173 | 0.966 |
| CO_2_^d^ (kgCO_2_) | 0.367 | 0.434 | 0.322 |
| CH_4_^n^ (kgCH_4_) | 0.021 | 0.025 | 0.019 |
| CO_2_^o^ (kgCO_2_) | 0.033 | 0.039 | 0.029 |
| **CO_2_ emitted (kgCO_2_)** | **0.340** | **0.473** | **0.351** |
| **CH_4_ emitted (kgCH_4_)** | **0.0213** | **0.025** | **0.019** |
| **GHG emissions (kgCO_2_ eq)** | **1.04** | **1.230** | **0.913** |

*Adapted from Benavides et al (2020).^6^

**Biodegradation percentages have been adapted from Afshar et al. (2024)^15^. The authors report that starch degrades at a rate approximately twice as low in landfills as in composting conditions. The same ratio (a division by two) was assumed for the polymers considered in this study.

[CH_4_] (%): CH_4_ concentration from decomposition of biodegradable plastic in landfill

CH_4_ oxidation (%): CO_2_ from the oxidation of non-captured CH_4_

CO_2_^s^: CO_2_ absorbed by biomass used for plastic production that remains as carbon in soil

CO_2_^d^: CO_2_ absorbed by biomass used for plastic production that ends up being degraded as CO_2_

CH_4_^n^: non-captured and non-oxidized CH_4_ emission

CO_2_^o^: CO_2_ absorbed by biomass used for plastic production that ends up being oxidized

# **Supplementary Table 11 | Parameters and assumptions used to estimate greenhouse gas emissions from composting of bio-based plastic packaging (1 kg)**

|  | **PLA** | **PHB** | **TPS** |
| --- | --- | --- | --- |
| Formula | C_3_H_4_O_2_ | C_9_H_14_O_4_ | C_6_H_12_O_5_ |
| MM_polymer_ | 72 | 194 | 164 |
| Carbon content | 0.5 | 0.56 | 0.44 |
| CO_2_ uptake | 1.83 | 2.04 | 1.61 |
| % biodeg (100yrs)** | 80 | 85 | 80 |
| [CH_4_] (%) | 5 | 5 | 5 |
| CH_4_ oxidation (%) | 36 | 36 | 36 |
| CO_2_^s^ (kgCO_2_) | 0.367 | 0.306 | 0.322 |
| CO_2_^d^ (kgCO_2_) | 1.394 | 1.648 | 1.223 |
| CH_4_^n^ (kgCH_4_) | 0.017 | 0.020 | 0.015 |
| CO_2_^o^ (kgCO_2_) | 0.026 | 0.031 | 0.023 |
| **CO_2_ emitted (kgCO_2_)** | **1.420** | **1.680** | **1.247** |
| **CH_4_ emitted (kgCH_4_)** | **0.017** | **0.020** | **0.015** |
| **GHG emissions (kgCO_2_ eq)** | **1.932056** | **2.285** | **1.696** |

*Adapted from Benavides et al (2020).^6^

**Biodegradation percentages have been adapted from Afshar et al.(2024)^15^. Given the high PLA content of the TPS, its biodegradation rate was assumed to be equivalent to that of PLA.

[CH_4_] (%): CH_4_ concentration from decomposition of biodegradable plastic in composting

CH_4_ oxidation (%): CO_2_ from the oxidation of non-captured CH_4_

CO_2_^s^: CO_2_ absorbed by biomass used for plastic production that remains as carbon in soil

CO_2_^d^: CO_2_ absorbed by biomass used for plastic production that ends up being degraded as CO_2_

CH_4_^n^: non-captured and non-oxidized CH_4_ emission

CO_2_^o^: CO_2_ absorbed by biomass used for plastic production that ends up being oxidized

# **Supplementary Table 12 | Parameters and assumptions used to estimate greenhouse gas emissions from marine littering of plastic packaging (1 kg)**

|  | **PLA** | **PHB** | **TPS** | **PET** | **PE** | **PP** | **PS** | **PVC** | **PUR** |
| --- | --- | --- | --- | --- | --- | --- | --- | --- | --- |
| Formula | C_3_H_4_O_2_ | C_9_H_14_O_4_ | C_6_H_12_O_5_ | C_10_H_8_O_4_ | C_2_H_4_ | C_3_H_6_ | C_8_H_8_ | C_2_H_3_Cl | C_15_H_10_N_2_0_2_ |
| MM_polymer_ | 72 | 194 | 164 | 192 | 28 | 42 | 104 | 62 | 250 |
| Carbon content | 0.5 | 0.56 | 0.44 | 0.63 | 0.86 | 0.86 | 0.92 | 0.39 | 0.72 |
| Potential CO₂ eq of carbon stock | 1.83 | 2.04 | 1.61 | 2.29 | 3.14 | 3.14 | 3.38 | 1.42 | 2.64 |
| % biodeg (100yrs)** | 3.4 | 100 | 3.4 | 12.2 | 5.9 | 38.9 | 1.26 | 0.4 | 9.6 |
| CO_2_^mw^ (kgCO_2_) | 1.77 | 0 | 1.55 | 2.01 | 2.96 | 1.92 | 3.34 | 1.41 | 2.39 |
| CO_2_^d^ (kgCO_2_) | 0.062 | 2.04 | 0.055 | 0.28 | 0.19 | 1.22 | 0.04 | 0.01 | 0.25 |
| **GHG emissions (kgCO_2_ eq)** | **0.062** | **2.04** | **0.055** | **0.28** | **0.19** | **1.22** | **0.04** | **0.01** | **0.25** |

*Adapted from Benavides et al. (2020)^6^.

**Biodegradation have been adapted from data reported by Corella-Puertas et al. (2023)^16^.

CO_2_^mw^: Carbon remaining in marine environment (CO₂ eq)

CO_2_^d^: CO₂ emitted from biodegradation

# **Supplementary Table 13 | Parameters for the characterization of microplastics impacts in LCA**

| **Polymer** | **Stage** | **Type of loss** | **Loss rate into the environment**^17^ | **Final release rate into ocean**^17^ | **Type of MPs released** | **MPs Size - Shape** | **Endpoint CFs – infinite – (PDF.m².yr/kg in the ocean)**** | **Endpoint CFs – short term – (PDF.m².yr/kg in the ocean)**** |
| --- | --- | --- | --- | --- | --- | --- | --- | --- |
| PLA | Polymer prod | Micro | 0.01% | 2% | Primary (MP_1_) | 1000 µm - Sphere | 8.45E+03 | 5.32E+03 |
|  | EOL | Macro | 1% | 5% | Secondary (MP_2_)* | 100 µm - Sphere | 4.30E+03 | 3.46E+03 |
| PHB | Polymer prod | Micro | 0.01% | 2% | MP_1_ | 1000 µm - Sphere | 9.44E+01 | 9.25E+01 |
|  | EOL | Macro | 1% | 5% | MP_2_* | 100 µm - Sphere | 7.00E+00 | 6.88E+00 |
| TPS | Polymer prod | Micro | 0.01% | 2% | MP_1_ | 1000 µm - Sphere | 8.45E+03 | 5.32E+03 |
|  | EOL | Macro | 1% | 5% | MP_2_* | 100 µm - Sphere | 4.30E+03 | 3.46E+03 |
| PET | Polymer prod | Micro | 0.01% | 2% | MP_1_ | 1000 µm - Sphere | 5.88E+03 | 4.11E+03 |
|  | EOL | Macro | 1% | 5% | MP_2_* | 100 µm - Sphere | 1.87E+03 | 1.62E+03 |
| HDPE | Polymer prod | Micro | 0.01% | 14% | MP_1_ | 1000 µm - Sphere | 6.58E+03 | 1.55E+03 |
|  | EOL | Macro | 1% | 5% | MP_2_* | 100 µm - Sphere | 1.34E+03 | 7.87E+02 |
| LDPE | Polymer prod | Micro | 0.01% | 14% | MP_1_ | 1000 µm - Sphere | 4.40E+03 | 1.37E+03 |
|  | EOL | Macro | 1% | 5% | MP_2_* | 100 µm - Sphere | 7.43E+02 | 5.20E+02 |
| PVC | Polymer prod | Micro | 0.01% | 2% | MP_1_ | 1000 µm - Sphere | 1.01E+04 | 5.77E+03 |
|  | EOL | Macro | 1% | 5% | MP_2_* | 100 µm - Sphere | 8.97E+03 | 5.54E+03 |
| PP | Polymer prod | Micro | 0.01% | 14% | MP_1_ | 1000 µm - Sphere | 1.59E+03 | 1.03E+03 |
|  | EOL | Macro | 1% | 5% | MP_2_* | 100 µm - Sphere | 1.32E+02 | 1.31E+02 |
| PS | Polymer prod | Micro | 0.01% | 14% | MP_1_ | 1000 µm - Sphere | 1.12E+04 | 1.77E+03 |
|  | EOL | Macro | 1% | 5% | MP_2_* | 100 µm - Sphere | 4.09E+03 | 1.45E+03 |
| EPS | Polymer prod | Micro | 0.01% | 17% | MP_1_ | 1000 µm - Sphere | 1.46E+03 | 3.60E+02 |
|  | EOL | Macro | 1% | 5% | MP_2_* | 100 µm - Sphere | 1.45E+02 | 1.12E+02 |
| PUR (adapted from Polyamide) | Polymer prod | Micro | 0.01% | 2% | MP_1_ | 1000 µm - Sphere | 6.30E+03 | 4.47E+03 |
|  | EOL | Macro | 1% | 5% | MP_2_* | 100 µm - Sphere | 2.17E+03 | 1.96E+03 |

*Secondary microplastics generated from macroplastic fragmentation (0.5% rate assumed).

**Characterization factors (CFs) for assessing the physical impact of microplastic emissions on marine biota in the infinite and short term (100 years) in PDF.m².yr/kg emitted (Potentially Disappeared Fraction of species per square meter after one year for one kg of plastic emitted). Adapted from Maga et al.(2022)^18^ and Saadi et al. (2025)^19^.

# **Supplementary Table 14 | Assumptions used to assess geographical variability in feedstock and energy for polymer production**

| **Scenario group** | **Type of feedstock** | **Location of feedstock production*** | | **Location of energy production*** | |
| --- | --- | --- | --- | --- | --- |
| PLA | Corn | US | market group for maize grain \| US | GLO | market group for electricity, low voltage \| GLO |
|  | Sugarcane | BR-MT | sugarcane production \| BR-MT | GLO | market group for electricity, low voltage \| GLO |
|  | Corn stover | US | market group for maize grain \| US | GLO | market group for electricity, low voltage \| GLO |
| PLA-low | Corn | US-IA | market for maize grain \| US-IA | RER | market group for electricity, low voltage \| RER |
|  | Sugarcane | CO | sugarcane production \| CO | RER | market group for electricity, low voltage \| RER |
|  | Corn stover | US-IA | market for maize grain \| US-IA | RER | market group for electricity, low voltage \| RER |
| PLA-high | Corn | RoW | market for maize grain \| RoW | CN | market group for electricity, low voltage \| CN |
|  | Sugarcane | IN | sugarcane production \| IN | CN | market group for electricity, low voltage \| CN |
|  | Corn stover | RoW | market for maize grain \| RoW | CN | market group for electricity, low voltage \| CN |

# **Supplementary Table 15 | Plastics demand in the European Union***

| **Polymer type** | **Packaging demand (EU) in 2020 (kt)** | **Packaging demand (EU) in 2050 with 3% growth (kt)** | **Packaging demand (EU) in 2050 with 3% degrowth (kt)** |
| --- | --- | --- | --- |
| PE | 9963 | 24183 | 3995 |
| PP | 4665 | 11323 | 1870 |
| PET | 3962 | 9616 | 1589 |
| PS | 1173 | 2847 | 470 |
| PVC | 459 | 1114 | 184 |

*adapted from Cimpan et al.(2021)^2^.

# **Supplementary Table 16 | Index**

| **Abbreviation** | **Explanation** |
| --- | --- |
| R_Conv_ | Conversion rate of polymer resin into packaging |
| R_Litt, prod_ | Release rate of microplastics to the environment |
| R_Inci_ | Incineration rate |
| R_Land_ | Landfilling rate |
| R_Comp_ | Composting rate |
| R_Litt, EOL_ | Release rate of macroplastics to the environment |
| R_Coll_ | Collection rate |
| R_Rec_ | Recycling rate |
| R_Rec, CL_ | Closed-loop recycled content rate |
| Bio-PE | Biobased Polyethylene |
| Bio-PET | Biobased Polyethylene Terephthalate |
| HDPE | High Density Polyethylene |
| LDPE | Low Density Polyethylene |
| PE | Polyethylene |
| PET | Polyethylene Terephthalate |
| PHB | Polyhydroxybutyrate |
| PLA | Polylactic Acid |
| PP | Polypropylene |
| PUR | Polyurethane |
| TPS | Thermoplastic Starch |
| PVC | Polyvinyl Chloride |
| GLO | Global |
| RoW | Rest of World |
| CO | Colombia |
| US | United States |
| US-IA | United States - Iowa |
| BR-MT | Brazil – Mato Grosso |
| RER | Europe |
| EOL | End-of-life |

# **Supplementary Methods**

Supplementary equations for calculating finite-horizon time fate matrices:

Within each compartment, the fate of particles undergoing first-order mass loss due to degradation and transfer processes can be expressed as the time integral of the remaining mass. Over an infinite time horizon, this integration yields:

$$\begin{aligned} \int_{0}^{\infty} m\left( t \right)dt= \int_{0}^{\infty} {(m}_{0}e^{-k_{tot}t})dt= \frac{m_{0}}{k_{tot}}=m_{0}\tau\#\left( 1 \right) \end{aligned}$$

**With,**

$m_{0}:$ **initial mass in the compartment**

$k_{\mathrm{tot}}$: sum of the degradation and transfer rates applied to particles emitted in the compartment

$\tau:$ residence time of the particle in the compartment

Over a finite time horizon, the expression becomes:

$$\begin{aligned} \int_{0}^{t} m\left( t \right)dt=\frac{m_{0}}{k_{tot}}\left( 1-e^{-k_{tot}t} \right)=\int_{0}^{\infty} m\left( t \right)dt\left( 1-e^{-k_{tot}t} \right) \#\left( 2 \right) \end{aligned}$$

This implies that over a finite time horizon, only a fraction of the total burden is captured $\left( 1-e^{-k_{tot}t} \right)$. The higher the value of $k_{tot}$ (rapid loss processes), the closer this fraction approaches 1, meaning that a 100-year horizon is sufficient to integrate almost the entire impact. Conversely, when $k_{tot}$ is small (highly persistent plastics), the fraction remains low, and a 100-year horizon substantially underestimates the long-term impact**.**

By applying this principle to interacting processes within a coupled multicompartment framework, the fate matrix ($FF$) over a time $T$ can be computed as follows:

$$\begin{aligned} FF = \int_{0}^{T} e^{KT}dT=-K^{-1}\left( I-e^{KT} \right) \#\left( 3 \right) \end{aligned}$$

**With,**

$K:$ **rates matrix regrouping degradation and transfer rates [yrs-1]**

Supplementary equations for calculating substitution trajectories:

$$\begin{aligned} Q_{Prim}= Q_{Conv}-Q_{Rec, CL} \#\left( 4 \right) \end{aligned}$$

$$\begin{aligned} Q_{Conv}=\frac{D}{R_{conv}}\#\left( 5 \right) \end{aligned}$$

$$\begin{aligned} Q_{Use}= D \#\left( 6 \right) \end{aligned}$$

$$\begin{aligned} Q_{Land}= D \times R_{Land}\#\left( 7 \right) \end{aligned}$$

$$\begin{aligned} Q_{Inci}= D \times R_{Inci} \#\left( 8 \right) \end{aligned}$$

$$\begin{aligned} Q_{Comp}= D \times R_{Comp} \#\left( 9 \right) \end{aligned}$$

$$\begin{aligned} Q_{Litt, prod}= D \times R_{Litt, prod}\#\left( 10 \right) \end{aligned}$$

$$\begin{aligned} Q_{Litt, EOL}= D \times R_{Litt, EOL}\#\left( 11 \right) \end{aligned}$$

$$\begin{aligned} Q_{Coll}= D \times R_{Coll} \#\left( 12 \right) \end{aligned}$$

$$\begin{aligned} Q_{Rec}= Q_{Coll}\times R_{Rec} \#\left( 13 \right) \end{aligned}$$

$$\begin{aligned} Q_{Rec, CL}=Q_{Rec}\times R_{Rec, CL} \#\left( 14 \right) \end{aligned}$$

**With,**

**Q denotes quantities of material flows (see Supplementary Fig.3), and D denotes total demand (see Supplementary Table 15).**

**R_x_ represents the conversion and EOL parameters (see Supplementary Tables 1 and 2).**

# **Supplementary References**

1. Zheng, J. & Suh, S. Strategies to reduce the global carbon footprint of plastics. *Nat. Clim. Change* **9**, 374–378 (2019).

2. Cimpan, C., Bjelle, E. L. & Strømman, A. H. Plastic packaging flows in Europe: A hybrid input‐output approach. *J. Ind. Ecol.* **25**, 1572–1587 (2021).

3. eunomia. *HDPE and PP Market in Europe, State of Play*. (2020).

4. ICIS. *PET Market in Europe, State of Play*. (2022).

5. Vural Gursel, I. *et al.* Comparative cradle-to-grave life cycle assessment of bio-based and petrochemical PET bottles. *Sci. Total Environ.* **793**, 148642 (2021).

6. Benavides, P. T., Lee, U. & Zarè-Mehrjerdi, O. Life cycle greenhouse gas emissions and energy use of polylactic acid, bio-derived polyethylene, and fossil-derived polyethylene. *J. Clean. Prod.* **277**, 124010 (2020).

7. Chen, L., Pelton, R. E. O. & Smith, T. M. Comparative life cycle assessment of fossil and bio-based polyethylene terephthalate (PET) bottles. *J. Clean. Prod.* **137**, 667–676 (2016).

8. García-Velásquez, C. & Van Der Meer, Y. Can we improve the environmental benefits of biobased PET production through local biomass value chains? – A life cycle assessment perspective. *J. Clean. Prod.* **380**, 135039 (2022).

9. Harding, K., Dennis, J., Vonblottnitz, H. & Harrison, S. Environmental analysis of plastic production processes: Comparing petroleum-based polypropylene and polyethylene with biologically-based poly-β-hydroxybutyric acid using life cycle analysis. *J. Biotechnol.* **130**, 57–66 (2007).

10. Gastaldi, E. *et al.* Degradation and environmental assessment of compostable packaging mixed with biowaste in full-scale industrial composting conditions. *Bioresour. Technol.* **400**, 130670 (2024).

11. Quantis and EA. *Plastic Leak Project - Methodological Guidelines V1, Vol. 3*. (2020).

12. Loubet, P., Couturier, J., Horta Arduin, R. & Sonnemann, G. Life cycle inventory of plastics losses from seafood supply chains: Methodology and application to French fish products. *Sci. Total Environ.* **804**, 150117 (2022).

13. Vink, E. T. H. & Davies, S. Life Cycle Inventory and Impact Assessment Data for 2014 Ingeo^TM^ Polylactide Production. *Ind. Biotechnol.* **11**, 167–180 (2015).

14. Ioannidou, S. M. *et al.* Techno-economic risk assessment, life cycle analysis and life cycle costing for poly(butylene succinate) and poly(lactic acid) production using renewable resources. *Sci. Total Environ.* **806**, 150594 (2022).

15. Afshar, S. V., Boldrin, A., Astrup, T. F., Daugaard, A. E. & Hartmann, N. B. Degradation of biodegradable plastics in waste management systems and the open environment: A critical review. *J. Clean. Prod.* **434**, 140000 (2024).

16. Corella-Puertas, E., Hajjar, C., Lavoie, J. & Boulay, A.-M. MarILCA characterization factors for microplastic impacts in life cycle assessment: Physical effects on biota from emissions to aquatic environments. *J. Clean. Prod.* **418**, 138197 (2023).

17. Hamonized methodology for assessing plastic leakage and impact. *Plastic Footprint Network* https://www.plasticfootprint.earth/assessment-methodology/.

18. Maga, D. *et al.* Methodology to address potential impacts of plastic emissions in life cycle assessment. *Int. J. Life Cycle Assess.* **27**, 469–491 (2022).

19. Saadi, N., Lavoie, J., Fantke, P., Redondo-Hasselerharm, P. & Boulay, A.-M. Including impacts of microplastics in marine water and sediments in life cycle assessment. *J. Clean. Prod.* **520**, 146037 (2025).
